# QuickStats: Percentage* of Adults Aged ≥45 Years Who Have Ever Had Lung Cancer,^†^ by Education Level — National Health Interview Survey,^§^ United States, 2021

**DOI:** 10.15585/mmwr.mm7145a6

**Published:** 2022-11-11

**Figure:**
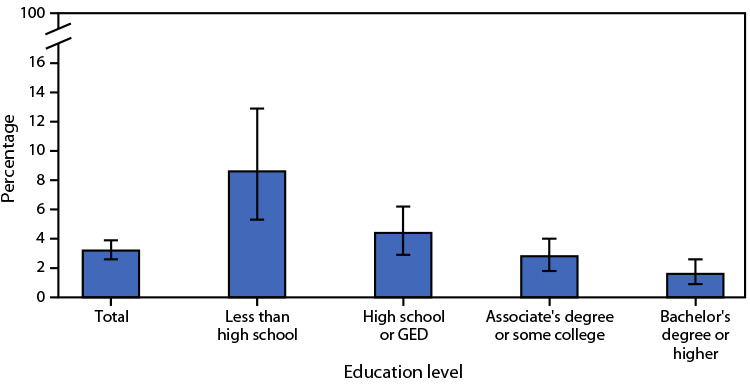
In 2021, 3.2% of adults aged ≥45 years had ever been told they had lung cancer. The prevalence of lung cancer among adults aged ≥45 years was highest for those with less than a high school education (8.6%). The percentage of adults who had ever had lung cancer decreased with increasing education level, with the lowest prevalence occurring among those with a bachelor’s degree or higher (1.6%).

**Source:** National Center for Health Statistics, National Health Interview Survey, 2021. https://www.cdc.gov/nchs/nhis.htm

For more information on this topic, CDC recommends the following link: https://www.cdc.gov/cancer/lung/basic_info/

